# Synthesis of Structurally Precise Polysiloxanes via the Piers–Rubinsztajn Reaction

**DOI:** 10.3390/ma12020304

**Published:** 2019-01-18

**Authors:** Xunjun Chen, Minghao Yi, Shufang Wu, Lewen Tan, Xin Ge, Ming He, Guoqiang Yin

**Affiliations:** 1 College of Chemistry and Chemical Engineering, Zhongkai University of Agriculture and Engineering, Guangzhou 510225, China; MHYi0848@163.com (M.Y.); SFWu2018@163.com (S.W.); williamrayz1996@gmail.com (L.T.); 18011779939@163.com (X.G.); heming1026@163.com (M.H.); yingq007@163.com (G.Y.); 2 Guangzhou key Laboratory for Efficient Utilization of Agricultural Chemicals, Guangzhou 510225, China

**Keywords:** Piers–Rubinsztajn (PR) reaction, silicone materials, morphological structure, composite structure, biomass-silicone composites

## Abstract

Silicone materials are widely used, from daily life to the military industry. With the advancement of science and technology and the increasing demands of industry, the requirement for high-performance precise structural silicone materials has increased. Therefore, the most important aspect in this field is finding a breakthrough in the synthetic methods. In this review, the latest research developments in controllable morphological structure and composite structure optimized synthesis of silicone materials using the Piers–Rubinsztajn (PR) reaction are summarized. The advantages of the PR reaction compared with traditional synthetic routes to silicone materials are presented. The highly controllable spatial structure of silicone materials and the structural combination of biomass or inorganic materials with silicone materials results in an improvement in performance or function. The morphological control of more complex silicone materials and the synthesis of non-traditional silicone materials with composite structures through the PR reaction will be the main research directions for the development of silicone materials in the future.

## 1. Introduction

Polysiloxanes are polymers containing ~R_2_SiO~ repeating units, which have Si–O bonds with a high bond energy (~ 530 kJ·mol^−1^) [1] and variable side groups on the main chain. This endows them with a very low surface energy, high thermal stability, a good gas barrier property, a low dielectric property, and therefore, good insulation, and excellent biocompatibility [2]. Since the commercial production of polysiloxane materials in 1940, they have gradually occupied an important position in the chemical, mechanical, electronics, and even aerospace industries. Polysiloxanes have many different forms; mainly linear, cyclic, branched, or 3D structures, however, it is quite difficult to control the structure and reactivity of such silicone-based polymers and materials [3]. The hydrolysis/dehydration of chlorosilanes or alkoxysilanes, or the base- or acid-catalyzed ring-opening polymerization of cyclic siloxane monomers such as cyclotetrasiloxane represent typical methods for the preparation of the siloxane backbone of polymeric silicone materials (see below) [1,4,5]. However, these approaches are not well suited to the synthesis of structurally precise polysiloxanes [6].

Because the chemical nature of Si is similar to that of C as they belong to the same family, silicones have created a semi-inorganic world of their own and were related to the carbon organics but are still destined to be different. Unlike the hydrogen in alkanes, the Si–H groups are hydrides [7]. Its reactivity has led to the emergence of many synthetic methods for controllable polysiloxanes, such as the hydrosilylation reaction [8]. Moreover, the strong Lewis acid catalyst tris(pentafluorophenyl)borane (B(C_6_F_5_)_3_; BCF), which was only used in the field of organic synthesis, was found to activate Si–H bonds for many reduction reactions [9,10]. In the 21st century, with Piers [10] and Rubinsztajn [11] pioneering the application of BCF in the field of catalyzed organosilicon synthesis, the development of structurally controlled polysiloxanes entered a new chapter. The polycondensation reaction between the hydrosilanes, or hydrosiloxanes, and oxygen-based nucleophiles [12] containing -OH groups or alkoxy groups is known as the Piers–Rubinsztajn (PR) reaction, in which BCF activates the Si–H bonds first.

## 2. Advantages of the PR Reaction over Traditional Synthetic Methods 

**Figure 1:**
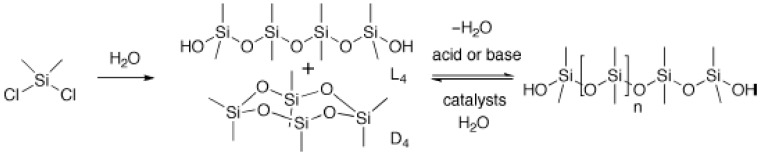
Conventional silicone hydrolysis polymerization synthesis route used in industry (Reproduced from Reference [2] with the permission).

The synthesis of silicones began with the nucleophilic substitution of water with chlorosilanes to produce linear and cyclic products with low molecular weights [2], as shown in Figure 1. These products are ultimately processed into high-molecular-weight functional materials, a process typically involving a nucleophilic substitution reaction catalyzed by acid or base. The disadvantage of this method is that it causes strong acid or base contamination and produces silanols as by-products, which leads to a decrease in the degree of crosslinking and interferes with the polymer structure. Even worse, in a strong acid or base system with the presence of water, even high-molecular-weight polysiloxanes undergo degradation, resulting in a broad molecular weight distribution of the polymers. For these reasons, it is particularly difficult to assemble polysiloxanes with complex structures reliably. 

Dehydrogenation coupling between organohydrogensilanes and organohydrosilanols is a convenient method for synthesizing well-defined polysiloxanes owing to high selectivity and easily removable hydrogen by-products [13]. The appearance of this method indicated that the synthesis of polysiloxanes was no longer uncertain. Further, metal-catalyzed directional polymerization emerged; however, such reactions require relatively high concentrations (~0.1 mol %) of precious metal catalysts such as platinum, palladium, rhodium, or ruthenium [14,15,16]. In addition, it has some unsatisfactory aspects in that only medium-molecular weight polymers are achieved, and uncontrolled silanol self-condensation occurs, which leads to complete alternation of the polymer structure [17,18].

As with the above process, the PR reaction catalyzed by BCF, the “metal-free” catalyst [19], can also be used to synthesize well-designed structured polysiloxanes. Such reactions are usually carried out under strictly controlled anhydrous conditions, avoiding structural defects and unintended side reactions caused by the presence of water [20,21]. In addition, the PR reaction has many other advantages, including high efficiency, easy removal of gas by-products, mild reaction temperature, low catalyst concentration, and easy control of polymer structure, which provide a new path for complex structured polysiloxanes [22] that are not available by conventional methods. Surprisingly, based on the wide range of oxygen-based nucleophiles (e.g., cellulose and biophenol extracted from nature), the PR reaction makes it possible to combine silicones with other substances (see below). Thus, new types of polysiloxanes breaking the traditional impression of mankind are introduced one after another.

## 3. Morphology-Controlled Synthesis of Polysiloxanes via the PR Reaction

### 3.1. Synthesis of Linear Polysiloxanes

**Figure 2:**
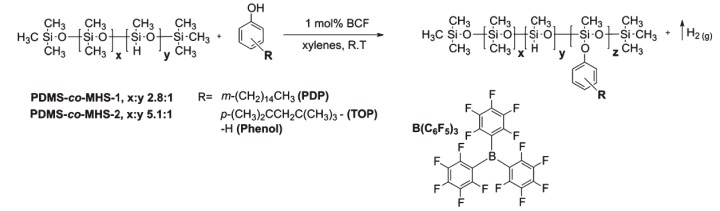
Synthesis of linear phenoxy-substituted polysiloxanes by the Piers–Rubinsztajn (PR) reaction (Reproduced from Reference [12] with the permission).

Linear polysiloxanes are usually oily liquids. Due to the stability, rotation, and flexibility of the Si–O bonds in the main chain with hydrophobic side groups, they have wide applications from high-temperature lubricants to cosmetic additives, which make them omnipresent in today’s society [12]. By varying the side groups of the linear polysiloxanes, many different properties can be achieved. Szawiola et al. [12] functionalized two poly(dimethylsiloxanes/methylhydrosiloxanes) (PDMS-*co*-MHS) using three different phenol derivatives (3-pentadecylphenol, 4-tert-octylphenol, and phenol) via the PR reaction to synthesize six new linear phenoxylated polysiloxanes, as shown in Figure 2. The appearance of these polymers varies from waxy solids to liquids, while different phenoxy segments directly affect their physical and thermal properties, as shown in Table 1. Among them, the thermal performance of the tert-octylphenoxy-substituted polymer is especially improved. For example, the 5% weight loss temperature of the TOP:PDMS-*co*-MHS 1 is 87.86% higher than that of the unmodified PDMS-*co*-MHS-1. The relaxation enthalpy of the PDP:PDMS-*co*-MHS-1, calculated based on the integrated area under the Differential Scanning Calorimeter (DSC) curve of the glass transition, is higher than that of the TOP:PDMS-*co*-MHS-2. Since the pentadecyl alkyl chain fragment is more strongly related to molecular motion and mutual attraction than the tert-octyl chain, more energy is required for the state transition. This review presents the effect of the introduction of a rigid phenoxy structure on the physical state and thermal properties of linear polysiloxanes. However, under BCF catalysis, the Si–H bond will undergo further reaction (metathesis reaction) with other nucleophilic oxygen atoms in the polymer backbone, resulting in an unintended broad distribution of molecular weight. Further, there was a rare high concentration for the amount of catalyst used, which did not meet the general condition of the PR reaction. 

**Figure 3:**
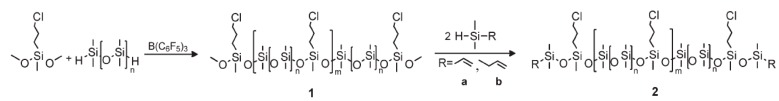
Synthesis of chloropropyl linear polysiloxanes by the PR reaction (Reproduced from Reference [13] published by The Royal Society of Chemistry).

In addition to improving the performance by modifying the side groups, the synthesis of linear silicone prepolymers with reactive side groups is also a major direction for such research. Madsen et al. [13] used the PR reaction between hydrogen-terminated polydimethylsiloxane (PDMS) and 3-chloropropylmethyldimethoxysilane to form a clear chain-shape silicone oil with chloropropyl groups. To eliminate the methoxy group at both ends of the long chain, vinyl or allylmethylsilane is used as a blocking agent for the next PR reaction. Finally, an alkenyl-terminated polyfunctional linear polysiloxane with chloropropyl groups distributed on the straight chain is obtained, which has further processing reactivity, as shown in Figure 3. This process is carried out at room temperature, gives a yield of up to 95% and a molecular weight of up to 70.3 kg·mol^−1^, and requires a low concentration of catalyst (<0.5 mol %).

**Figure 4:**
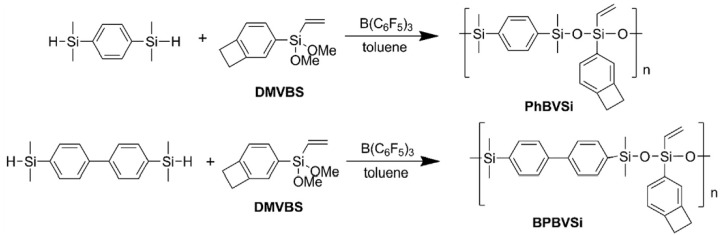
Synthesis of benzocyclobutene (BCB) oligomers by the PR reaction [26]. Reproduced with the copyright permission.

However, linear polysiloxanes are susceptible to thermal degradation under high-temperature conditions due to the presence of pure Si–O–Si bonds formed by the rearrangement/equilibrium of siloxane chains. The low glass transition temperatures owing to the highly flexible chains may limit their use as engineering materials requiring high strength. As described below, the replacement of Si–O repeating units has become a new idea for designing new linear silicone materials. The introduction of silphenylene units into the polysiloxane backbone can significantly improve their thermal stability, radiation resistance, and mechanical property [23,24,25]. By the PR reaction, a benzocyclobutene (BCB)-functionalized silphenylene-siloxane oligomer (PhBVSi) can be produced by polycondensation between 1,4-bis(dimethylsilyl)benzene and (1,2-dihydrobenzocyclobuten-4-yl)dimethoxy(vinyl)silane (DMVBS), and a BCB-functionalized silbiphenylene-siloxane oligomer (BPBVSi) can be produced by polycondensation between 4,4′-bis(dimethylsilyl)bibenzene and DMVBS, as shown in Figure 4 [26]. The molecular weights of these two polysiloxanes are not particularly large and their polydispersities are not broad (PhBVSi:Mw = 4829 g·mol^−1^, Polydisperity (PDI) = 1.60; BPBVSi:Mw = 10,571 g·mol^−1^, PDI = 1.57). The vinyl and four-membered rings they carry can undergo other cycloaddition reactions (non-PR processes) and curing, endowing the two resins with expected thermal stability (5% thermal degradation weight loss temperature is 500 °C and 491 °C, respectively). In addition, no glass transition was detected from room temperature to 350 °C, indicating that the introduction of phenylene and biphenylene structures greatly increase the rigidity of such linear polysiloxanes. This work has led to the synthesis of rigid linear silicone oligomers by the PR reaction followed by addition crosslinking to obtain a stronger resin, although the steps are complicated. 

### 3.2. Synthesis of Cyclic Polysiloxanes

**Figure 5:**
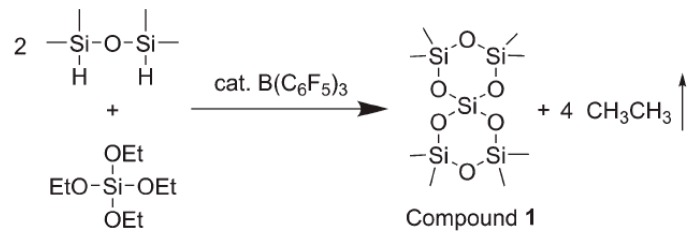
Synthesis of small molecule cyclic siloxane by the PR reaction [31]. Reproduced with the copyright permission.

Compared to linear polymers, cyclic polymers have a unique topological structure and therefore own some interesting physical properties [27,28]. For example, the incorporation of ring-stress constrained cyclotetrasiloxane into a polymer results in the development of a variety of functional materials, including thin film electrolytes [29] and self-healing materials [30]. Therefore, the cyclic polymers in the field of silicone materials should be an unmissable topic for researchers. Liu et al. [31] initially attempted to synthesize a small molecule with a bicyclic ring under BCF catalysis using 1,1,2,2-tetramethyldisiloxane and tetraethoxysilane, as shown in Figure 5. 

**Figure 6:**
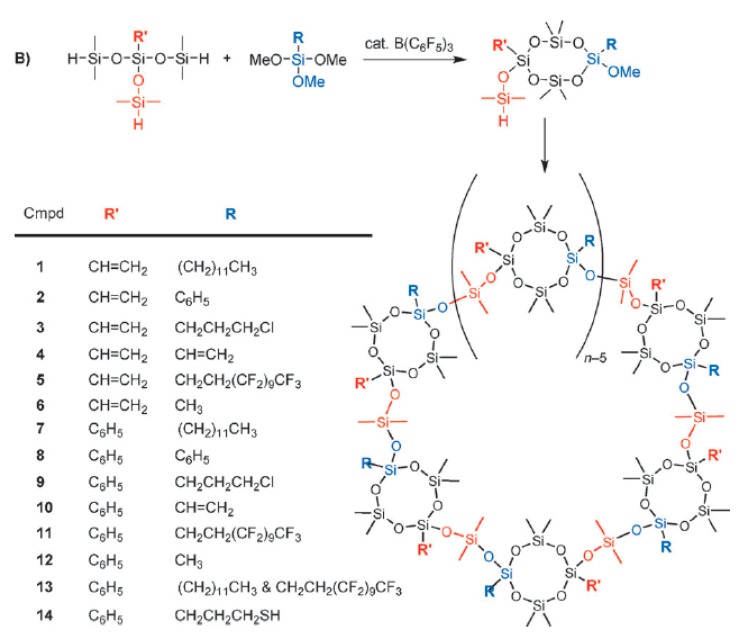
Synthesis of cyclic polysiloxanes by the PR reaction (Reproduced from Reference [32] with the permission).

This small molecule, Compound 1 in Figure 5, underwent ring-opening polymerization to further yield macromolecular cyclic polymers although the path involved was not a PR reaction. However, this indicated the introduction of the PR reaction in the field of cyclic polysiloxanes. In the subsequent study, Liu et al. [32] were committed to synthesizing true cyclic polysiloxanes by a one-step PR reaction. When a hydrosiloxane and an alkoxysilane are rapidly dropped into the BCF catalytic system, a “ring-and-loop” cyclic polysiloxane is obtained, as shown in Figure 6. When the side groups on the ring were vinyl and dodecyl, the molecular weight could reach 305.0 kg·mol^−1^ and the polydispersity index is as high as 3.61, which might be caused by intermolecular coupling during the reaction. By controlling the drop rate, a polydispersity index of 1.16 could be achieved for the polymers with the phenyl side groups and the perfluorodecylethyl side groups, while the molecular weight was as high as 6.7 kg·mol^−1^. Some of the cyclic polymers even achieved the possibility of directing the assembly of the nanoparticles. For example, HAuCl_4_ is reduced with NaBH_4_ in the presence of polymer 14. The topological structure of these polymers could be verified by conventional characterization methods such as Si nuclear magnetic resonance (^29^Si-NMR) spectroscopy. Interestingly, these macromolecular rings were clearly visible under an atomic force microscope (AFM).

### 3.3. Synthesis of Bridge-link-type Polysiloxanes

**Figure 7:**
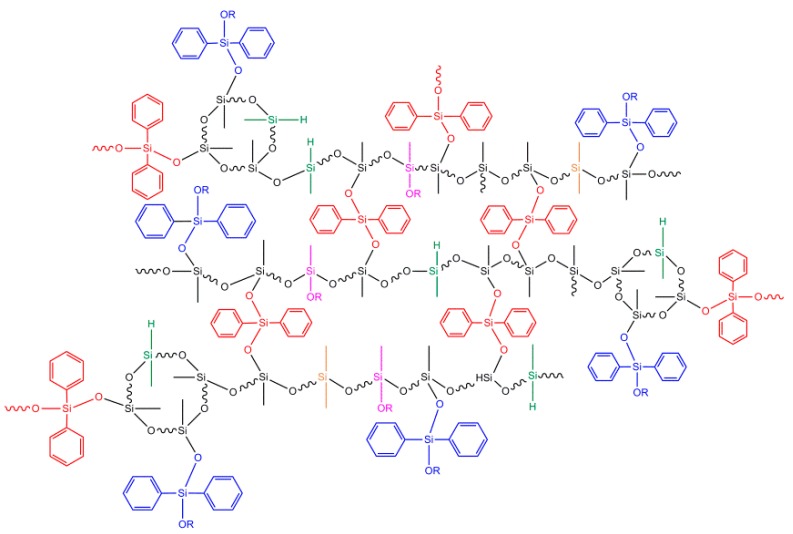
Complex bridge-link-type polysiloxanes (Reproduced from Reference [33]).

Silicone materials, whether linear or cyclic, have excellent properties. Thus, what kind of performance would the polysiloxanes possessing linear structures and molecular bridges similar to macrocyclic structures exhibit inside molecules? Considering this idea, the authors’ research group [33] used the PR process to polycondense high hydrogen-containing oligosiloxanes and diphenyldialkoxysilane to obtain complex bridge-like-type polysiloxanes possessing both phenyl substituents and diphenyl siloxy molecular bridges [(C_6_H_5_)_2_Si(OSi)_2_], as shown in Figure 7. This material has excellent thermal stability (about 70% residual mass at 700 °C in nitrogen atmosphere) and high refractive index, and retains two active reactive groups, Si–H groups and alkoxy groups, as well. It can be used as a good addition-type crosslinking agent with adhesion-promoting properties or as a special curing agent for curing a silicone material by addition and condensation reaction simultaneously, having a potential application in the light-emitting diode (LED) packaging industry. The reaction proceeds under mild and green conditions (optimum temperature: 0–25 °C; catalyst concentration: 0.8 mmol·L^−1^). However, the process has drawbacks, such as frequent exchange reactions of Si–H and alkoxy groups at high temperatures, formation of small ring structures with a high ring-stress effect, and easy gelation caused by excessively crosslinking in a high reactant concentration (50%, 65%). Nevertheless, this is a new idea for the preparation of a new bridge-link structure of polysiloxanes. Following our work, Tian et al. [26] also prepared similar highly crosslinked polysiloxanes: linear polysiloxanes with benzocyclobutene groups were first synthesized by a PR reaction and then crosslinked by a cycloaddition reaction. However, our one-step PR reaction is more efficient and convenient.

## 4. Synthesis of Biomass-Structure Polysiloxanes via the PR Reaction

Silicone materials are important commercial materials that affect the daily lives of developed economies [34]. In fact, they are quite environmentally friendly. Numerous studies have shown that under environmental and biological-mediated pre-depolymerization and oxidation, almost all silicones are easily decomposed into sand, water, and CO_2_ [35]. However, due to their synthetic pathways and refractory properties, they have less chance of belonging to “Green Chemistry”. Fortunately, the PR reaction has provided a mild, efficient, and green synthetic pathway. To change the traditional impression of silicone materials, researchers are interested in not only seeking breakthroughs in the morphology of silicone polymers, but also introducing easily degradable green biomass structures into silicone materials to develop new materials that are consistent with the concept of sustainable development.

### 4.1. Synthesis of Lignin-Silicone Composites

**Figure 8:**
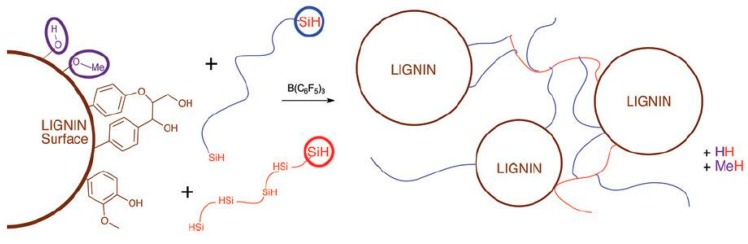
Scheme synthesis of lignin-silicone composites by the PR reaction [37]. Reproduced with the copyright permission.

Zhang et al. [36] reacted 1,1,1,2,2-pentamethyldisiloxane with lignin under BCF catalysis to achieve the reduction and degradation of hardwood and softwood lignin. Because the functional groups and the complex network structure of these two lignins are very different, the degrees of reduction and degradation are not the same. Hardwood lignin can be reduced to a good extent with a reduction conversion rate of 95% at a concentration of 10 wt % BCF. On the contrary, since many bonds from the coniferyl alcohol units of softwood lignin are not easily affected by the reaction, softwood lignin is more susceptible to surface modification by the silane groups, and the solubilization of degradation product was limited to about 30 wt %. Based on this difference, Zhang et al. further tried to prepare softwood-lignin-silicone crosslinked materials by using the PR reaction [37]. After quantifying the reactive groups on the surface of the softwood lignin particles, the BCF catalyst and different kinds of hydrosiloxanes were added to the system, and finally, a softwood-lignin-silicone elastomer was obtained by the PR process. Since the PR process is a polycondensation reaction accompanied by a gas (hydrogen or lower alkane) overflow, the resulting elastomer may actually be a porous foam material or a biomass-silicone rubber in case the crosslinking was too fast for the gas to escape. The latter requires a finely controlled depressurization venting step. It can be seen from Table 2 (selected parts) that the optimum amount of lignin is 41 wt %, and the elastomer obtained under this condition exhibits good tensile properties. This indicates that a high content of biomass in materials does not degrade the mechanical properties; however, the content should avoid being too low or too high. Because lignin is difficult to process due to its network structure [36], it is naturally degraded in nature [38] and is regarded as an inexpensive fuel in industry [39]. The use of the PR reaction to crosslink softwood lignin with hydrosiloxanes to prepare greener silicone rubbers not only avoids the waste of lignin resources, but also realizes the combination of silicone and biomass in structural designs, as shown in Figure 8. 

### 4.2. Synthesis of Eugenol-silicone Composites

Eugenol is an organic substance that can be easily extracted from cloves and other plants [40]. Compared to lignin having a number of functional groups and complex network structures, eugenol is a small monomer having only three different functional groups. Owing to the different activity of different functional groups, macromolecules with different structures can be synthesized selectively. Based on the selectivity of the PR reaction toward the phenolic hydroxyl group and the phenylalkoxy group of the eugenol molecule, a platinum-catalyzed hydrosilation reaction was used to selectively synthesize the allyl group to form an extended-chain polymer or elastomer containing the embedded biomass structure, or foam, in which the structure and constituent parts of the material can be manipulated at will [41]. Eugenol-based macromonomers, linear polymers, functional telechelic siloxanes, block siloxane copolymers with molecular weight up to 660 kg·mol^−1^, elastomers as well as foams can be prepared by a simple conversion of different telechelic hydrogen-terminated siloxane oligomers and reaction sequences. The eugenol-modified polysiloxane exhibits superior hydrolysis resistance similar to that of phenoxysilane due to space and electronic effects, with only 1.3–1.5% weight loss rate after soaking in boiling water for 12 h. The research work realized the introduction of eugenol, a biomass raw material that is readily available in nature, into siloxanes, whose synthesis process is both efficient and precisely controllable. However, the shortcoming is that BCF would be affected by the degradation of the platinum catalyst or weak poisoning. To ensure the progress of the reaction need adding amount of BCF, while the platinum catalyst is not affected by BCF.

Compounds with aryl structures have always attracted the widespread attention of academia and industry because they can be converted into high-performance polymers possessing high thermal stability, low water absorption, and good dielectric property [42,43,44]. As a phenol, eugenol also has an aryl structure. Based on the above research, new functional materials based on introduction of eugenol into silicone materials were created. BCB hydrosilane and eugenol were reacted via the PR process to successfully prepare a novel silicone prepolymer based on biomass [45]. The prepolymers were solidified by a Diels–Alder cycloaddition reaction at a high temperature due to the BCB units, and the obtained eugenol-silicone composites exhibited various advantageous properties. The good overall properties impart huge advantages to the material compared to other materials, as shown in Table 3, which render the material promising for potential application in the microelectronic industry, such as encapsulating resins for integrated circuit (IC) dies, or laminated matrix resins for manufacturing printed circuit boards. This significant work not only combined eugenols and silicones to synthesize high-performance green silicone materials that are a breakthrough in the traditional concept of polysiloxanes, but also achieved high value-added utilization of biomass raw materials.

## 5. Synthesis of Inorganoparticle-Silicone Polymers via the PR Reaction

Inorganic particles generally do not have multiple or diverse reactive functional groups like biomass materials do, which limits their covalent attachment to silicones. Therefore, in most cases, inorganoparticles are only used as filler materials to mix with polysiloxanes to form composite materials. If the inorganoparticles carry oxygen-based nucleophilic groups after functionalization, the PR reaction will break the deadlock and realize inorganoparticle-silicone covalently bonded materials. 

### 5.1. Synthesis of Carbon Nanotube-silicone Materials

**Figure 9:**
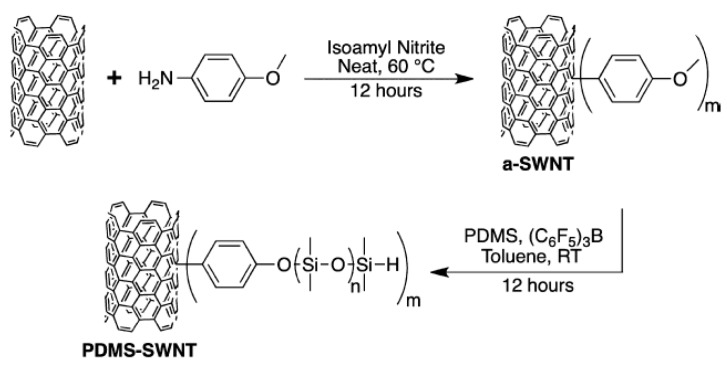
Preparation of polydimethylsiloxane (PDMS)-SWNT by PR reaction after functionalization of single-walled carbon nanotubes (SWNTs) [53]. Reprinted with permission from *Macromolecules* **2014**, *47*, 6527–6530. Copyright 2014 American Chemical Society.

Carbon nanotube (CNT)-silicone materials have several potential applications, including healing or charge-dissipating elastomers [46], biocompatible and flexible electrodes or sensors [47], lithium-ion batteries [48], strong elastomers [49], capacitive energy harvesting [50], skin-like stretch sensors [47,51], and other electronic products [52]. After surface functionalization of the CNTs, the PR reaction can be used to covalently bond the CNT to a linear hydrogen-terminated PDMS, as shown in Figure 9 [53]. Owing to such graft modification, the CNT-silicone materials are soluble in polar organic solvents due to the good solubility of polymethylsiloxanes in most organic solvents (solubility in 1,2-dichloroethane: 273 mg·L^−1^, solubility in tetrahydrofuran: 358 mg·L^−1^), subverting the property of traditional CNT materials as insolubles. The increase in solubility greatly enhances the processing prospects of CNT in the polymer field, which may help incorporate CNTs into PDMS elastomers, including rubber and foam. Compared with the conventional CNT-silanization reaction in which only surface modification is implemented (including the use of a large amount of halo- or alkoxysilane to react with the surface of the CNT [54,55]), the PR reaction fills the gap in the preparation process of the CNT-silicone composite polymers. However, this method has several limitations. When the amount of added CNT is 2–5 wt %, its dispersion in the reactant system is not effective and uniform catalyst mixing cannot be achieved, which results in uncontrolled inconsistent crosslinking.

### 5.2. Synthesis of Graphene Oxide-Silicone Materials

Graphene oxides (GO) have attracted the attention of researchers worldwide owing to their remarkable electrical, heat-conducting, and mechanical properties [56,57,58]. To take advantage of these properties, the use of GO in combination with different polymeric matrices has proven to significantly improve the performance of various polymer composites [59,60,61,62,63]. Zhang et al. [64] had successfully incorporated GO into the PDMS chain by a simple PR reaction. The material was readily incorporated into the bulk silicone elastomer as a reinforcing additive in concentrations ranging from 1 to 10 wt %. When the addition amounts were 5 and 10 wt %, the mechanical properties (approximately 100% increase in elongation at break) and oxygen barrier property of the GO-PDMS elastomer improved, as shown in Table 4. This work demonstrated that the PR reaction is a promising strategy for the preparation of various GO-loaded elastomers and functional materials. However, addition of a high amount of GO to increase performance (~5–10%) is not economical and more in-depth research is required to optimize the results.

## 6. Conclusions

Traditional synthesis of polysiloxanes through the hydrolysis polymerization method has several problems, such as strong acid and alkali pollution and uncontrollable polymer structure. Moreover, the dehydrogenation coupling and hydrosilylation reactions catalyzed by precious metal catalysts cannot avoid shortcomings such as heavy metal pollution, unexpected by-products, relatively large catalyst consumption, and low economic efficiency. In this regard, since its inception in the 21st century, the PR reaction has been widely used in the field of silicone synthesis and favored by researchers worldwide owing to its mild reaction conditions, high efficiency, high selectivity of catalysis, and non-polluting characteristics. In addition, the PR reaction exhibits a high degree of morphological control during silicone polymerization that cannot be achieved by conventional synthetic processes, and can even introduce biomass or inorganic structures from other sources into the polysiloxanes, thereby breaking through the boundaries of traditional silicone materials and achieving performance improvements. It is worth noting that the PR process requires precise control as it has the problem of rapid reaction and easy gelation as well as easy metathesis at high temperatures. The vision of achieving more beautiful morphological structural polysiloxanes or more non-traditional silicone composite materials through the PR reaction is not a distant future.

## Figures and Tables

**Table 1:** Summary of thermal properties of all phenoxylated polymers and the unmodified base polymers (Summarized from Reference [12]).

Polymers	*T*_5%_/°C	*T*_g/_°C	Δ*H*_relaxation_/J·g^−1^
PDMS-*co*-MHS-1	140	-	-
0.75:1 ^a^ PDP:PDMS-*co*-MHS-1	177	45-50	28.5
0.75:1 ^a^ TOP:PDMS-*co*-MHS-1	263	-	-
0.75:1 ^a^ phenol:PDMS-*co*-MHS-1	152	-	-
PDMS-*co*-MHS-2	150	-	-
0.75:1 ^a^ PDP:PDMS-*co*-MHS-2	244	-	-
0.75:1 ^a^ TOP:PDMS-*co*-MHS-2	130	75-80	2.8
0.75:1 ^a^ phenol:PDMS-*co*-MHS-2	150	-	-

Note: ^a^ The molar ratio of the raw materials in the polymer. *T*_5%_ denotes 5% thermal degradation weight loss temperature. *T*_g_ denotes the glass transition temperature.

**Table 2:** Mechanical properties of lignin-silicone composites (Summarized from Reference [37]).

Lignin-Silicone Elastomer (LE-^#^)	Shore OO	Shore A	Modulus/MPa	Elongation at Break/%
LE-0.5	35–45	-	-	-
LE-27	85–90	33–35	0.93 ± 0.22	286 ± 40
LE-41A	85–90	35–40	1.31 ± 0.02	260 ± 14
LE-41B	85–90	45–50	3.28 ± 0.39	146 ± 17
LE-41F	85–90	45–50	1.95 ± 0.49	297 ± 30
LE-41G	70–75	20–25	0.15 ± 0.01	305 ± 21
LE-66	85–90	75–85	-	-

Note: “#” in “LE-#” indicates the amount of lignin added (in wt %).

**Table 3:** Performance comparison of benzocyclobutene-silicone-eugenol resin (BCB-Si-E) with other commercial resins (Summarized from Reference [45]).

Samples	Dielectric Constant (D_k_)	Thermostability	Water Uptake (%)	Storage Modulus (GPa)
Cured BCB-Si-E	2.77	*T*_5%_ of 400 °C	0.15	5.9
Commercial bisphenol A-type epoxy resin	- ^a^	- ^a^	1.25	- ^a^
Anethole-based BCB resin	2.9	- ^a^	- ^a^	- ^a^
Polybenzoxazine resins	2.81	- ^a^	- ^a^	- ^a^
BCB resins	2.8	- ^a^	- ^a^	- ^a^
Softwood-lignin-based polycarbonates	- ^a^	*T*_5%_ of 346 °C	- ^a^	- ^a^
Softwood-lignin-based cyanate esters	- ^a^	*T*_5%_ of 375 °C	- ^a^	- ^a^

Note: *T*_5%_ denotes 5% thermal degradation weight loss temperature. ^a^ The value is not set as a comparison.

**Table 4:** Mechanical properties and oxygen permeability of graphene oxide (GO)-reinforced silicones (Summarized from Reference [64]).

Sample	Percentage of GO-PDMS (wt %)	Break Strength (MPa)	Elongation at Break (%)	Oxygen Permeability Coefficient (mm·L·m^−2^ Day·Bar)
Control	0	0.38 ± 0.09	103 ± 9	250 ± 0.6 %
1	1	0.27 ± 0.04	79 ± 11	273 ± 0.4 %
2	3	0.19 ± 0.02	144 ± 29	220 ± 0.4 %
3	5	0.51 ± 0.04	247 ± 38	193 ± 0.5 %
4	10	0.48 ± 0.22	202 ± 76	135 ± 0.6 %
